# The Oncogenic and Tumor‐Suppressive Roles of SNHG18: A Double‐Edged Long Noncoding RNA in Cancer

**DOI:** 10.1155/bmri/9651227

**Published:** 2026-07-03

**Authors:** Vida Dehghani Champiri, Samira Asgharzade

**Affiliations:** ^1^ Department of Student Research Committee, Shahrekord University of Medical Science, Shahrekord, Iran, skums.ac.ir; ^2^ Department of Cellular and Molecular Research Center, Basic Health Science Institute, Shahrekord University of Medical Science, Shahrekord, Iran, skums.ac.ir

**Keywords:** cancer, lncRNAs, *SNHG18*

## Abstract

Numerous recent studies have shown that long noncoding RNAs (lncRNAs) play a crucial role in the development of diverse cancers. Small nucleolar RNA host gene 18 (*SNHG18*) is a cancer‐related lncRNA in the SNHG family, whose expression is dysregulated in various tumors and acts as either an oncogenic or tumor‐suppressive factor. Several studies have demonstrated that SNHG18 exhibits dual effects in various cancers. The role of this molecule is context‐dependent, acting as a tumor suppressor in certain cancers while exhibiting oncogenic properties in others. The expression of *SNHG18* is cancer‐type specific: it is upregulated in glioma, multiple myeloma, and lung cancer, but downregulated in bladder and hepatocellular cancer. Clinical studies have also found a correlation between *SNHG18* expression and prognosis in different cancers. We present an overview of studies on SNHG18, emphasizing its biological functions and its potential as a promising biomarker for the diagnosis and treatment of diverse cancer types.

## 1. Introduction

More than 200 different types of cancer are recognized [1]. Cancer arises from the combined effects of genetic alterations—such as mutations—and epigenetic changes. Factors that affect the incidence and progression of cancer have been linked to protein‐encoding regions, as 80% of the human genome is transcribed into RNA, and the majority of this RNA is noncoding [2–4].

There are two groups of RNAs: coding and noncoding RNAs (ncRNAs). ncRNAs, which do not encode proteins, include a diverse range of molecules such as ribosomal RNAs (rRNAs), transfer RNAs (tRNAs), microRNAs (miRNAs), long noncoding RNAs (lncRNAs), small nuclear RNAs (snRNAs), and small nucleolar RNAs (snoRNAs) [5]. LncRNAs are a heterogeneous group typically defined as transcripts longer than 200 nucleotides, accounting for over half of the noncoding RNA in mammals. They take part in various biological processes, such as transcriptional gene modulation, conservation of genomic unity, inactivation of the X chromosome, genomic imprinting, cell differentiation, and progression. LncRNAs have gained much attention in the community because of their role in cell proliferation, transformation, and tumorigenesis in different cancer signaling and cancer phenotypes. Accumulating evidence suggests that lncRNAs have modulated expression and may act as regulators of coding genes in the development of various cancers [6–9].

*SNHGs*, as a small nucleolar RNA host gene, contain introns and exons, and the intron regions are converted into snoRNAs. SNHGs can regulate DNA methylation, transcription, and translation in cells [5, 10]. *SNHGs* have been described to be upregulated in various cancers and may influence cell proliferation, apoptosis, invasion, migration, drug resistance, and other cancerous functions. Molecularly, SNHGs are competing endogenous RNAs (ceRNAs) [11]. ceRNAs sponge miRNA and impact the binding of other transcription factors, thereby regulating gene expression [12]. For example, SNHG6 enhances EZH2 expression by sponging miR‐26a/b and miR‐214 in colon cancer [13]. The SNHG family consists of SNHG1–SNHG20 [5].

*SNHG18* is part of the SNHG family located on Chromosome 5p15.31 and has three exons [5, 10, 14]. *SNHG18* is in the nucleus and cytoplasm and can regulate the expression of DNA. Studies have shown that SNHG18 has double‐edged roles in various cancers. For instance, it promotes epithelial–mesenchymal transition (EMT), enhances tumor cell invasion and migration, regulates cytoskeletal regeneration, and influences cell motility in glioma, while exerting a suppressive effect on liver cancer [15]. In this survey, we reviewed the oncogenic and tumor‐suppressive impact of the molecular mechanism of SNHG18 on various cancers.

## 2. Materials and Methods

This literature was provided through searches of major databases, including PubMed, Scopus, and Web of Science, for articles published up to 2024. Search terms included “*SNHG18*,” “lncRNA,” “long noncoding RNA,” and cancer‐related keywords such as “tumor,” “carcinoma,” “glioma,” and “metastasis.” Relevant original studies were screened based on their relevance to the role of *SNHG18* in cancer biology and clinical implications.

## 3. Function of SNHG18 as an Oncogene in Human Cancers

### 3.1. Nonsmall Cell Lung Cancer (NSCLC)

Corresponding to the Global Cancer Statistics in 2020, lung cancer is the cause of 1.8 million cancer deaths worldwide [16]. Lung cancer usually exists in two forms: one form is NSCLC, and the other form is small cell lung cancer (SCLC) [17, 18]. NSCLC accounts for the majority of lung cancer. NSCLC is categorized into squamous‐cell carcinoma, adenocarcinoma, and large‐cell carcinoma subtypes [19].

Abundant lncRNAs are overexpressed and function as oncogenes in NSCLC to promote processes like proliferation, survival, invasion, migration, and metastasis to become cancer. For instance, Metastasis‐associated lung adenocarcinoma transcript 1 (MALAT1) is an lncRNA that is upregulated in NSCLC, or NEAT1enhances NSCLC cell proliferation and migration by sponging miR‐181a‐5p. In contrast, some lncRNAs act as tumor suppressors, like growth arrest‐specific 5 (GAS5) by regulating p53, p21, and E2F1, which prevent tumor proliferation and induce apoptosis in NSCLC [20–22].

In their study on the mechanism of SNHG18 on NSCLC, Fan et al. identified *SNHG18* as a regulator circuit of a transcription factor, Megakaryocytic leukemia‐1 (MKL1). They demonstrated that MKL1 promotes *SNHG18* expression, which in turn drives oncogenic processes. Moreover, *SNHG18* depletion significantly suppressed NSCLC tumorigenesis and metastasis in vivo and in vitro studies, underscoring its oncogenic function. The tumor‐enhancer effects of SNHG18 were largely mediated through its antagonistic interaction with miR‐211‐5p, which resulted in the upregulation of bromodomain‐containing protein 4 (BRD4). The functional significance of this axis was confirmed by the fact that silencing BRD4 abrogated the proliferative and invasive phenotypes induced by *SNHG18*. These suggest that SNHG18 contributes to malignant progression in NSCLC through modulation of the miR‐211‐5p/BRD4 pathway. This led to cell proliferation and invasion, which caused NSCLC growth and metastasis [23, 24]. Furthermore, clinical data revealed that *SNHG18* expression was significantly higher in tumor tissues than in adjacent normal tissues, and patients with higher *SNHG18* levels showed lower overall survival (OS) [23].

### 3.2. Glioma Cancer

Glioma is one of the most dominant brain and spinal tumors, influencing men more than women [25]. There are different degrees of glioma, including Grades I, II, III, and IV, but only the III and IV groups cause death and severe complications [26]. A very small proportion of glioma cancers are associated with genetic disorders. Several risk factors, such as ionizing radiation exposure or allergy history, increase the risk of glioma [27, 28].

Numerous lncRNAs in glioma are linked to both metastatic tumor development and patient prognosis. They can also act as signaling mediators and regulators for the expression of several genes in this pathway [29]. Both CRNDE and FEZF1‐AS1 are well‐characterized oncogenic lncRNAs in glioma that function as ceRNAs, also known as miRNA sponges. CRNDE promotes tumor growth and migration by sequestering tumor‐suppressive miRNAs such as miR‐337‐3p and miR‐136‐5p, thereby upregulating downstream prooncogenic targets including ELMO domain‐containing protein 2 (ELMOD2) and B‐cell lymphoma 2 (BCL2). Similarly, FEZF1‐AS1 enhances glioma cell migration and invasion by sponging miR‐34a and miR‐363‐3p, leading to stabilization of NIN1/RPN12 binding protein 1 homolog (NOB1). Together, these lncRNAs drive malignant progression through ceRNA‐based mechanisms [30–32].

#### 3.2.1. Impact of SNHG18 on Invasion and Progression of Glioma Cells

Many lncRNAs are implicated in the regulation of EMT, and one of them is SNHG18, which induces perineural invasion of mesenchymal and epithelial cells and mesenchymal transition in glioma cells [7]. SNHG18 enhanced the expression of *β*‐catenin, N‐cadherin, vimentin, SLUG, SNAIL, and MMP‐9, which are involved in EMT. Additionally, SNHG18 contributes to glioma cell motility through the regulation of *α*‐enolase (ENO1, an enzyme located in the nucleus that has a role in proliferation and metastasis [33]) [34]. Clinical data further support this, demonstrating that elevated *SNHG18* expression in glioma patients correlates with enhanced EMT activity [35].

Beyond EMT, SNHG18 is associated with ferroptosis (a cell death factor that is iron‐dependent, regulated cell death [36]), by influencing the progression and metastasis pathways [37].

#### 3.2.2. *SNHG18* Promotes Radioresistance of Glioma Cells

Semaphorin 5A (*SEMA5A*) is one of the semaphorin families that play a crucial role in nervous system guidance [38, 39].

Previous studies have documented that *SNHG18* is located 125 bases upstream of the *SEMA5A*. Silencing *SNHG18* upregulates *SEMA5A* expression in glioma cell lines, revealing a negative correlation. Given that *SEMA5A* suppresses glioma cell migration, further experiments demonstrated that *SNHG18* depletion enhanced radiosensitivity, an effect that is attenuated upon *SEMA5A* knockdown. This indicates that the radiosensitization effects of *SNHG18* depletion are associated with *SEMA5A* expression. Specifically, following ionizing radiation, *SEMA5A* knockdown significantly decreased DNA damage response markers such as *γ*‐H2AX and caspase‐3, whereas deletion of *SNHG18* yielded the opposite results. Ultimately, these in vivo and in vitro findings demonstrate that SNHG18 might increases resistance to radiation therapy by inhibiting SEMA5A in glioma cells [40].

#### 3.2.3. Motility With the Interference of ENO1

SNHG18 is predominantly localized in the nucleus, so Zheng et al. speculated that it may promote glioma cell invasion via interaction with the nuclear proteins. One of these proteins is the enzyme ENO1, which contributes to cytoskeleton remodeling and the regulation of cell movement and migration. Silencing ENO1 expression with interfering RNA abolished the invasion induced by SNHG18 in glioma cells. Although SNHG18 did not alter ENO1 expression but instead suppressed the protein′s nucleocytoplasmic transport. These findings suggest that SNHG18 may regulate cell movement by disturbing the nucleocytoplasmic transport of ENO1, thereby promoting EMT and cytoskeletal remodeling [34].

#### 3.2.4. Mechanism of *SNHG18* on Glioma

Mechanistically, Early 2 factor (E2F, a family of transcription factors [41]) promotes the SNHG18 expression by binding to its promoter. Consistently, E2F expression was positively correlated with *SNHG18* levels, suggesting that this oncogenic transcription factor upregulates *SNHG18*. Furthermore, SNHG18 was shown to modulate miR‐211‐5p, thereby influencing downstream targets critical for cancer cell survival and proliferation. In addition, SNHG18 modulates forkhead box D1(FOXD1, which has an oncogene manner in different types of tumors [42]) positively by suppressing miR‐338‐5p (miR‐338‐5p is a conserved miRNA among mammals, which initially was identified as a differentially expressed gene [43]). These findings ascertained that SNHG18 may function as ceRNA for miR‐338‐5p, thereby facilitating the glioma cell proliferation through modulation of the axis miR‐338‐5p/FOXD1 [44].

Proteins such as N‐Cadherin, E‐Cadherin, and Vimentin are well‐established markers of the EMT process. By increasing the N‐Cadherin and Vimentin expression and inhibiting the E‐Cadherin expression, SNHG18 promotes EMT and may exerts a tumorigenic effect in glioma cells [35].

### 3.3. Multiple Myeloma (MM)

MM is a type of malignant plasma cell that originates from the bone marrow. In this disorder, an excess of immunoglobulin is produced [45]. The accumulation of this immunoglobulin and interaction with bone marrow cells causes problems such as anemia and bone lesions [46].

Several hematopoietic‐associated lncRNAs have been recognized that are involved in the proliferation and apoptosis of myeloma cells. OIP5‐AS1 (OIP5 antisense RNA 1) regulates the miR‐410 expression by inhibiting the effect of prooncogenic processes like cell growth and cell cycle progression. Abnormal expression of lncRNAs led to hematologic malignancies such as lymphomas, leukemias, and myeloma. These lncRNAs also promoted MM and resistance to anticancer drugs. As suggested, these lncRNAs in myeloma cancer can be utilized as biomarkers to diagnose and treat [47–51].

A study showed that SNHG18 was associated with increased *SEMA5A* expression in MM, and the cooverexpression of *SNHG18* and *SEMA5A* corresponded with a more aggressive disease phenotype and poorer OS, including a reduction in median OS. SNHG18 may be useful for the prognosis of MM cancer by oncogenic behavior [52].

### 3.4. Gastric Cancer (GC)

GC is one of the fifth most common malignant tumors in the world. The rate of GC among adults below 50 years has been increasing. Except for *Helicobacter pylori* infection, the occurrence of GC has been related to lifestyle and genetic factors [53, 54].

Several lncRNAs have an oncogenic role; the lncRNA CCHE1 escalates GC proliferation and progression. Some of the lncRNAs can act as tumor suppressors, like lncRNA SNHG1, which modulates the influence of DCLK1/Notch1 pathway on cell migration and EMT in GC by regulating miR‐15b [55].

A recent study in GC reported the SNHG18 as an enhancer of tumor growth and metastasis. The research elucidated a pathway where Src homology 2 domain containing phosphatase 2 (SHP2) activates the ROS/JNK/NFAT4 signaling pathway, resulting in elevated *SNHG18* expression. In addition, SNHG18 was shown to modulate miR‐211‐5p, thereby influencing downstream targets related to cancer cell survival and proliferation. These findings establish SNHG18 as an oncogene in GC. Clinically, high *SNHG18* expression is associated with poor prognosis, suggesting that *SNHG18* depletion could offer a new approach for promising treatment of gastric malignancy [56].

## 4. Function of SNHG18 as a Tumor Suppressor

### 4.1. Hepatocellular Carcinoma (HCC)

HCC or liver cancer is one of the fatal cancers, with a low incidence of survival and a poor prognosis [57–59]. HCC is the most prominent kind of liver cancer [60]. Factors that cause HCC include viral infections, the formation of nodules, and liver cirrhosis. Many lncRNAs expressed in HCC can act as a biomarker [61].

Some lncRNAs inhibit the migration, metastasis, and proliferation of liver cancer cells, whereas others have the opposite effect [62]. For instance, the lncRNA‐ubiquitin‐fold modifier conjugating enzyme 1 (MT1DP) induces cancer processes and inhibits apoptosis in HCC cells [41]. MT1DP regulates *α*‐fetoprotein (AFP, a marker for liver cancer) with negative feedback and diminishes protein synthesis of forkhead box A1 (FOXA1), which is a transcription factor in HCC growth and cancer proliferation [42]. In the molecular pathway, *SNHG18* might modulate the PIK3/AKT/mTOR signaling pathway, which is required for cell proliferation, growth, and survival. Because this pathway in HCC is dysregulated, targeting it could inform strategies for the treatment of liver cancer. Its expression is associated with tumor size. The aberrant lncRNA expression is associated with the malignancy of cancers, and it is no exception for HCC [63].

According to Liu et al.′s findings, the clinical and prognostic value of SNHG18 in HCC patients could demonstrate that the expression of *SNHG18* was considerably decreased in HCC tissues and plasma compared to normal liver tissues and cirrhosis. Furthermore, expression of *SNHG18* was found to correlate with the levels of AFP and tumor sizes. The study suggests that a combination of SNHG18 with AFP can be useful in diagnosing patients whose serum AFP levels are lower than the standard (200 ng/mL), and indicates that SNHG18 may act as a tumor suppressor in HCC [64].

#### 4.1.1. Hepatitis B Virus (HBV)–Associated With HCC

HBV infection increases the progression of the disease and causes liver cancer. Factors that cause HBV‐related HCC include HBV genome integration, genome instability due to a series of mutations, activation of signal pathways that stimulate oncogenesis, and cancer [65–67].

To investigate the impact of SNHG18 with oleanolic acid (OA), Song et al. utilized four HBV‐associated liver cancer cell lines and a normal hepatocellular cell line. OA is a five‐ring triterpenoid compound found in medicinal plants. This compound has an antitumor effect, which decreases the survival of liver cancer cells. Molecularly, SNHG18 potentiates the antitumorigenic effects of OA in HBV‐related HCC by suppressing key cancer processes, including invasion and proliferation, suggesting OA can modulate the expression of *SNHG18* for promising therapeutic potential in HBV‐related HCC. They also indicated that downregulation of *SNHG18* expression was significantly associated with elevated AFP levels and advanced tumor‐node‐metastasis (TNM) staging of HBV‐associated HCC patients, which suggests a crucial effect on key cellular processes. *SNHG18* expression was decreased in tumor tissues compared to normal tissues. The patients with low levels of this lncRNA exhibited lower overall survival. These findings underscore the potential of SNHG18 as a promising therapeutic target for HBV‐HCC [68].

### 4.2. Bladder Cancer (BC)

BC is an important cancer in the genitourinary system, and its incidence is increasing every year. Many lncRNAs are correlated to BC, like MeG3 and PTENP1. They suppress cell proliferation, metastasis, and invasion. In contrast, RP11‐89 and SNHG16 increase cell growth and migration [16, 69–72].

According to the report of Meixia Ke et al., *SNHG18* was significantly downregulated in BC tissues and cell lines when compared to normal bladder tissues. Mechanistically, SNHG18 was shown to suppress c‐Myc transcription factor by regulating the ubiquitin–proteasome pathway, thereby reducing c‐Myc levels. Downregulation of c‐Myc promotes transcription and accumulation of P21, an inhibitor of cyclin‐dependent kinase, leading to cell‐cycle arrest at the G0/G1 phase. Consequently, SNHG18 diminishes cell proliferation and cell growth in BC. Moreover, the tumor suppressor behavior of SNHG18 in BC suggests its prognostic value. Higher expression of *SNHG18* is correlated with improved OS rates, which can be a useful prognostic indicator for BC patients [73].

Collectively, these findings indicate that the double‐edged roles of SNHG18 across different cancer types may act as either oncogenic or tumor‐suppressor factors, depending on the tumor context. The functional characteristics of SNHG18 in various cancers are summarized in Table 1.

**Table 1:** Functional characteristics of SNHG18 in various cancers.

Cancer type	Sample size	Expression level	Roles	As a promising candidate biomarker	Genes/proteins related	Ref.
Hepatocellular carcinoma (HCC)	Preoperative samples of HCC: 80	Downregulated	↓Invasion	Validated with AFP	AFP, alanine aminotransferase, and aspartate aminotransferase	[(64)]
	Samples of cirrhosis: 83		↓Proliferation			
	Samples of chronic hepatitis: 60		↓Metastasis			
	Control tissue: 83		↓Migration			
Hepatitis B Virus‐Associated Hepatocellular Carcinoma (HBV‐HCC)	Four HCC cell lines with HBV transform: HepG2/2.2.15, SNU182, MHCC97H, and PLC8024	Downregulated	↓Invasion	Proposed with oleanoic acid	Not identified	[(68)]
	Normal liver cell: L02		↓Proliferation			
	Tumor tissues with high SNHG18: 53		↓Migration			
	Tumor tissues with low SNHG18: 58					
Bladder cancer (BC)	Cell lines of BC cancer: UMUC3, J82, RT4, RT112, TCCSUP, and T24	Downregulated	↓Proliferation	Proposed	P21	[(73)]
	BC tissues: 48 pairs				P27	
	Control tissues: 48 pairs (from same patient)				c‐Myc	
Nonsmall cell lung cancer (NSCLC)	NSCLC tissue from: 63	Upregulated	↑Proliferation ↑Colony formation ↑Migration	Proposed	MKL‐1	[(23)]
	Control tissue: 63					
	Five cell lines of NSCLC: A549, H1299, H23, H460, and H1792		↑Tumorigenesis		MiR‐211‐5p	
			↑Metastasis		BRD4	
Multiple myeloma (MM)	MM tissue: 47 newly diagnosed MM, 18 CR MM, and 13 refractory/relapse MM	Upregulated	↑Invasion	Proposed	SEMA5A	[(52)]
			↑Proliferation			
					P53	
	Control tissue: 22 with iron deficiency anemia					
Gastric cancer (GC)	GC cell line: MGC‐803 CAR‐T cells	Upregulated	↑Proliferation ↑Colony formation ↑Migration	Proposed	SHP2 ROS/JNK/NFAT4 signaling pathway miR‐211‐5p BRD4, PD‐L1, IDO1, Bax, Caspase‐3, and Caspase‐8	[(56)]
			↑Tumorigenesis			
			↑Metastasis			
Glioma cancer	Glioma tissue: 44	Upregulated	↑Migration	Proposed	MiR‐338‐5p	[(44)]
	Control tissue: not mentioned					
					FoxD1	
					E2F1	
			↑Invasion			
	5 cell lines of glioma cancer (U251,		↑Progression			
	U87, LN229, LN308, and T98G cells)					
	Glioma tissue: 44	Upregulated	↑Progression	Proposed	SEMA5A	[(40)]
	Control tissue: 70					
	Four cell lines of glioma cancer: MO59K, M059J, U87 and U251		↑Radioresistance			
	Three cell lines of glioma cancer: M059J, M059K, and U251	Upregulated	↑Invasion	Proposed	ENO1, *β*‐catenin, SNAIL, SLUG, Vimentin, N‐cadherin, MMP‐2, MMP‐9, and E‐cadherin	[(34)]
			↑Migration			

*Note:* Biomarker potential: “Validated” indicates clinical association confirmed in human cohorts; “proposed” indicates association in preclinical/small human studies.

## 5. Conclusion and Perspectives

Recently, various investigations into lncRNAs have revealed an association between the diagnosis and prognosis of cancers [74]. lncRNAs are implicated in various cell processes, including differentiation, proliferation, cell migration, etc. [74, 75].

SNHG18 is one of the SNHG family, which has recently attracted the attention of many scientists. Research experiments proposed that SNHG18 has context‐dependent roles in various cancers. *SNHG18* has high expression in cancers such as glioma, lung, gastric, and MM cancers, which can promote proliferation, invasion, EMT, and migration. Conversely, it has low expression in liver and BC, which can suppress proliferation, invasion, EMT, and migration. We suggest that this double‐edge behavior of SNHG18 may be because of its highly context‐dependent nature, and its function is influenced by tissue type, active signaling pathways, and the genetic and epigenetic status of cells. Also, this lncRNA is associated with the diagnosis and prognosis of these cancers; in that case, it might be used as a promising therapeutic target for different types of cancers.

Although numerous studies have investigated the SNHG18, further study is still required to understand its roles in diagnosis, prognosis, and as a promising biomarker. Moreover, evidence linking SNHG18 to certain cancers, such as prostate, breast, and ovarian, is limited, highlighting the necessity for more comprehensive studies in these areas.

## Funding

No funding was received for this manuscript.

## Conflicts of Interest

The authors declare no conflicts of interest.

## Data Availability

The data that support the findings of this study are available from the corresponding author upon reasonable request.
